# Ginseng Extract G115 Attenuates Ethanol-Induced Depression in Mice by Increasing Brain BDNF Levels

**DOI:** 10.3390/nu9090931

**Published:** 2017-08-24

**Authors:** Weerawan Boonlert, Hattaya Benya-aphikul, Jariya Umka Welbat, Ratchanee Rodsiri

**Affiliations:** 1 Department of Pharmacology and Physiology, Faculty of Pharmaceutical Sciences, Chulalongkorn University, Bangkok 10330, Thailand; one_tsuke@hotmail.com (W.B.); h.benyaaphikul@gmail.com (H.B.); 2 Department of Anatomy, Faculty of Medicine, Khon Kaen University, Khon Kaen 40002, Thailand; jariya@kku.ac.th

**Keywords:** *Panax ginseng*, ginseng extract G115, ethanol, depression, BDNF, mice

## Abstract

The decrease of brain-derived neurotrophic factor (BDNF) has been reported in alcohol use disorder and major depression. The effective treatment of these comorbid diseases remains undiscovered. Nutraceutical products are therefore proposed as an alternative approach to overcome this challenge. Ginseng extract G115, the standardized extract of *Panax ginseng*, is a widely-used nutraceutical that is beneficial for various central nervous system disorders. This study aimed to determine the antidepressant effect of ginseng extract G115 in ethanol-treated mice models. Mice received either water, amitriptyline, or various doses of G115 (p.o.) followed by water or ethanol (i.p.) for 8 days. The antidepressant activity was evaluated using forced swimming test. BDNF levels were measured from hippocampal and prefrontal cortex tissues. The results demonstrated that the increase of immobility time in depressant mice induced by ethanol was reversed by both G115 and amitriptyline treatment. A significant increase of BDNF levels in the hippocampus and prefrontal cortex was observed in ethanol-treated mice receiving G115. Taken together, this study provides scientific information on the use of G115 as an antidepressant that could be further used as a dietary supplement in comorbid alcohol use and major depressive disorders.

## 1. Introduction

Alcohol use disorder often occur with other psychiatric disorders, including major depression [1]. Comorbid alcoholism and depression cause increases of alcohol consumption and suicide rates [2,3]. Treatment of depression in alcohol use disorder patients is essential to support alcohol abstinence and alcohol relapse prevention [4]. Drugs used for the treatment of alcohol dependence, such as disulfiram and naltrexone, failed to alleviate depression symptoms in alcohol use disorder patients [5]. Antidepressants have been clinically used to treat depression symptoms in alcohol use disorder. To date, the efficacy of antidepressants in comorbid depression and alcoholism is modest and inconsistent, while there are high risks of adverse effects and drug interaction problems [6].

The similarities between pathology of alcohol dependence and depression have been observed [7,8]. Brain-derived neurotrophic factor (BDNF) has a major role in neurogenesis, neuronal plasticity, growth, and survival [9]. The reduction of BDNF levels in the hippocampus and prefrontal cortex contributed to various neuropsychiatric disorders, including mood disorders and addiction [10]. The reduction of serum BDNF was reported in comorbid alcohol dependence and depression patients [11]. In addition, the decreases of BDNF levels in the hippocampus and frontal cortex were previously reported in alcohol-treated rats [12].

Panax ginseng C.A. Meyer is a widely-used medicinal plant in Asia. It serves as an adaptogenic herb which helps increased body resistance to stress, trauma, anxiety, and fatigue. Ginseng extract G115 is the standardized extract of P. ginseng, which is extensively used as a nutraceutical in Asia and worldwide [13]. Previous studies using high-performance liquid chromatography (HPLC) and gas chromatography (GC) reported that ginseng extract G115 is composed of ginsenosides Rb1, Rb2, Rc, Rd, Re, Rg1, Rg2, Rf, and Ro [14]. The effects of P. ginseng and ginsenosides in the central nervous system disorders have been increasingly reported including their neuroprotective effects and their benefits in neuropsychiatric disorders [15]. Moreover, P. ginseng has a high safety profile with no, or little, drug interaction [13]. Therefore, the present study aimed to determine the effect of ginseng extract G115 in ethanol-induced depression mice and its underlying antidepressant mechanisms involving BDNF levels in the brain.

## 2. Materials and Methods

### 2.1. Drugs and Chemicals

Absolute ethanol (Macron Fine Chemical™, Center valley, PA, USA) was diluted in sterile water to the concentration of 38% v/v. Ethanol-treated mice were injected by 38% ethanol intraperitoneally in a constant volume of 10 mL/kg (equal to 3 g/kg ethanol).

Amitriptyline (Sigma-Aldrich, St. Louis, MO, USA) was dissolved in sterile water to the concentration of 1 mg/mL. Mice were given amitriptyline by oral gavage in a constant volume of 10 mL/kg.

Ginseng extract G115 used in this study is the standardized extract made from the roots of P. ginseng which adjusted to 4% ginsenosides (Ginsana^®^ SA, Bioggio, Switzerland). The extract, contained in soft gelatin capsules as 100 mg of ginseng extract G115 per capsule, was dissolved in sterile water to the concentration of 10, 20, 40, and 80 mg/mL. Mice were given ginseng extract G115 by oral gavage in a constant volume of 10 mL/kg.

### 2.2. Animals and Treatment

Male Imprinting Control Region (ICR) mice, weighing 20–25 g (National Laboratory Animal Center, Mahidol University, Bangkok, Thailand), were housed in groups of five per cage at a constant ambient temperature (25 ± 2 °C) and humidity (50–60%) under a 12-h light/dark cycle with free access to food and water. Animal experiments were carried out in accordance with the Ethical Principles and Guidelines for the Use of Animals for Scientific Purposes of the National Research Council of Thailand. All procedures were approved by the Institutional Animal Care and Use Committee of the Faculty of Pharmaceutical Sciences, Chulalongkorn University, Bangkok, Thailand (approval No. 14-33-003).

Mice were divided into 12 groups (*n* = 8/group) as indicated in Table 1. Water, amitriptyline, or G115 was given orally one hour before intraperitoneally injection of either ethanol or water. Treatments were given once daily for eight consecutive days. All mice were performed in the open field test followed by forced swimming test 1-day after the last treatment (experimental day 9). After behavioral testing, mice were euthanized with 100 mg/kg pentobarbital sodium (i.p., Nembutal^®^, CEVA Sante’animale, Brussels, Belgium). The brain was quickly removed. The prefrontal cortex and hippocampus were dissected and kept at −80 °C for later analysis of BDNF levels using western blot analysis.

### 2.3. Behavioral Testing

#### 2.3.1. Open Field Test

Locomotor activity of mice was tested in an open field arena to examine whether repeated ethanol and ginseng extract G115 administration affects mice motor activity. Mice were placed in a black square box (50 × 50 × 41 cm) for five minutes. Ambulation time was recorded and analyzed using VideoMOT2 (TSE systems, Bad Homburg, Germany).

#### 2.3.2. Forced Swimming Test

A forced swimming test [16] was performed one hour after the open field test. Mice were placed in a cylinder (height: 24 cm diameter: 10 cm) containing 15 cm of water at 25 ± 1 °C for six minutes. Mouse swimming behaviors were recorded using video camera for further analysis of immobility time and swimming time. Immobility time is the time that a mouse floats with small movement to maintain its head above the water level. Swimming time is the time that a mouse moves on the water surface in a horizontal position.

### 2.4. Western Immunoblotting

The method was modified from Umka et al. [17]. Briefly, mouse hippocampus and prefrontal cortex were homogenized in ice-cold lysis buffer and a cocktail of protease inhibitors. The homogenate was centrifuged at 13,000 rpm at 4 °C for 10 min and supernatant was collected. The protein concentrations were measured by Nanodrop 2000 spectrophotometer (Thermo Scientific, Waltham, MA, USA). Equal amounts of protein samples (80 µg) were separated by electrophoresis using 14% SDS-PAGE gels. The separated proteins were then transferred onto the nitrocellulose membrane. The membrane was blocked using blocking buffer (5% skim milk in BSA) for one hour at room temperature. Blots were probed with the following primary antibodies: polyclonal anti-BDNF (1:150 SantaCruz Biotechnology, Santa Cruz, CA, USA) and anti-GAPDH (1:20,000 Abcam, Cambridge, UK) overnight at 4 °C. The blot was incubated with secondary conjugated antibodies (anti-rabbit (1:2000) and anti-mouse (1:2000) IgG, HRP-linked antibody, Dako, Carpinteria, CA, USA) at room temperature for one hour. The blot was developed using the enhanced chemiluminescence method by a luminescent image analyzer (GE Healthcare Bio-Sciences, Uppsala, Sweden). The signal was visualized with ECL Western blotting substrate. Protein band densities were quantified with Image-J software and expressed as the density of BDNF/GAPDH.

### 2.5. Statistical Analysis

Statistical analysis was performed using Graphpad Prism 7.0 (GraphPad Software, Inc., San Diego, CA, USA). Data were analyzed using one-way analysis of variance (ANOVA) followed by Fisher’s LSD post-hoc test for multiple group comparison. *p* < 0.05 was considered as statistically significant.

## 3. Results

### 3.1. Forced Swimming Test

One-way ANOVA revealed the significant effect of treatment on immobility time (*F*(11,84) = 11.97, *p* < 0.0001) and swimming time (*F*(11,84) = 8.26, *p* < 0.0001) in the forced swimming test. 

**Figure 1:**
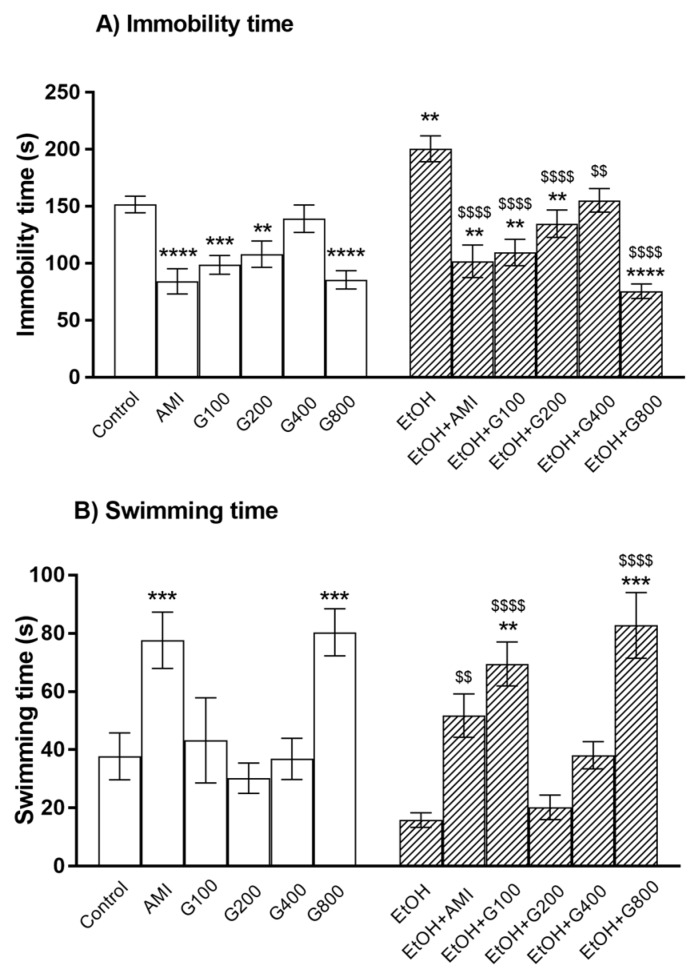
The immobility time (**A**) and swimming time (**B**) of mice in the forced swimming test. AMI, amitriptyline; EtOH, ethanol; G100, G200, G400, G800, ginseng extract G115 100, 200, 400, or 800 mg/kg, respectively. ** *p* < 0.01, *** *p* < 0.001, and **** *p* < 0.0001 compared to Control, ^$$^ *p* < 0.01 and ^$^^$$$^ *p* < 0.0001 compared to EtOH group.

As a positive control, amitriptyline significantly decreased immobility time and increased swimming time compared to controls (*p* < 0.0001 and *p* < 0.001, respectively) (Figure 1). Similarly, G115 (100, 200, and 800 mg/kg) treatment significantly decreased immobility time compared to controls (*p* < 0.001, *p* < 0.01, and *p* < 0.0001, respectively) (Figure 1A). G115 (800 mg/kg) treatment also increased swimming time compared to controls (*p* < 0.001) (Figure 1B). These results indicated an antidepressant-like effect of G115 in normal mice.

Repeated ethanol administration can induce depression in the force swimming test as ethanol treatment significantly increased immobility time compared to controls (*p* < 0.01) (Figure 1A). Amitriptyline showed the antidepressant-like effect in ethanol-treated mice as the lower immobility time and higher swimming time were presented in mice treated with amitriptyline plus ethanol. G115 also attenuated ethanol-induced depression as giving G115 (100, 200, 400, and 800 mg/kg) to ethanol-treated mice significantly decreased immobility time compared to ethanol-treated alone mice (*p* < 0.0001, *p* < 0.0001, *p* < 0.01, and *p* < 0.0001, respectively) (Figure 1A). In the same way, G115 (100 and 800 mg/kg) plus ethanol-treated group had significantly higher swimming time compared to ethanol-treated alone mice (*p* < 0.0001) (Figure 1B). Moreover, mice receiving G115 (100, 200, and 800 mg/kg) plus ethanol had significantly lower immobility time compared to controls (*p* < 0.01) and mice receiving G115 (100 and 800 mg/kg) plus ethanol had higher swimming time compared to control (*p* < 0.01 and *p* < 0.001, respectively), suggesting the potent antidepressant-like effect of G115.

### 3.2. Locomotor Activity

**Figure 2:**
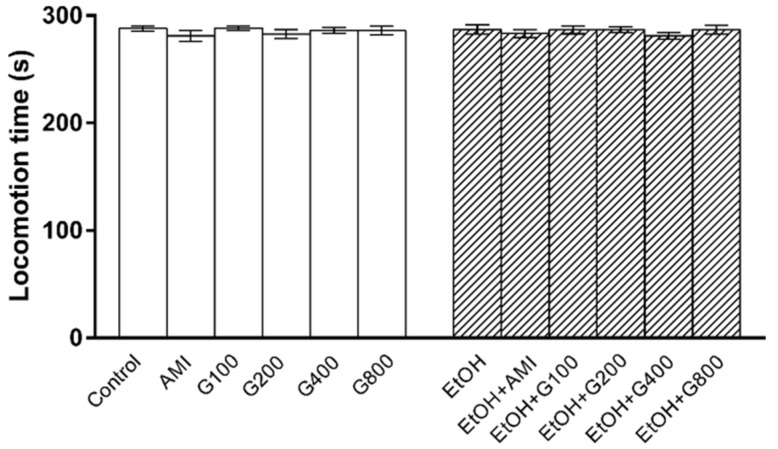
The locomotion time of mice in open field test. AMI, amitriptyline; EtOH, ethanol; G100, G200, G400, G800, ginseng extract G115 100, 200, 400, or 800 mg/kg, respectively.

Locomotor activity was performed to determine whether repeated administration of ethanol and G115 affect mice motor function, which would interfere swimming performance in the forced swimming test. There was no significant effect of treatment on locomotion time in this experiment (Figure 2) suggesting that an increase in swimming time was mainly due to an antidepressant-like effect of G115.

### 3.3. BDNF Levels in the Hippocampus

**Figure 3:**
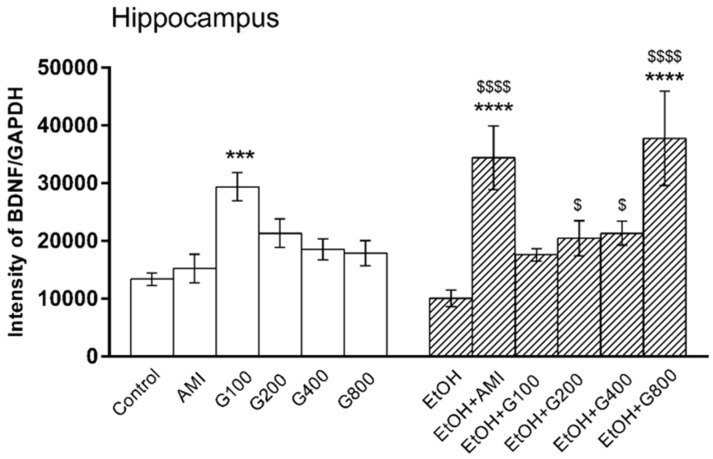
Effects of treatments on BDNF levels in the hippocampus. AMI, amitriptyline; EtOH, ethanol; G100, G200, G400, G800, ginseng extract G115 100, 200, 400, or 800 mg/kg, respectively. *** *p* < 0.001 and **** *p* < 0.0001 compared to Control, ^$^ *p* < 0.05 and ^$^^$$$^ *p* < 0.0001 compared to the EtOH group.

There was an overall effect of treatment on BDNF levels in the hippocampus (*F*(11,82) = 6.47, *p* < 0.0001). G115 (100 mg/kg) significantly increased hippocampal BDNF compared to controls (*p* < 0.001). Repeated ethanol administration tended to decrease BDNF levels in the mouse hippocampus, but this effect was not significantly different from controls. However, mice in amitriptyline plus ethanol and G115 (200, 400, and 800 mg/kg) plus ethanol had significant increases of hippocampal BDNF levels compared to ethanol-treated alone (*p* < 0.0001, *p* < 0.05, *p* < 0.05 and *p* < 0.0001, respectively). Moreover, mice treated with amitriptyline plus ethanol and G115 (800 mg/kg) plus ethanol had significantly higher levels of BDNF in the hippocampus compared to controls (*p* < 0.0001) (Figure 3).

### 3.4. BDNF Levels in the Prefrontal Cortex

**Figure 4:**
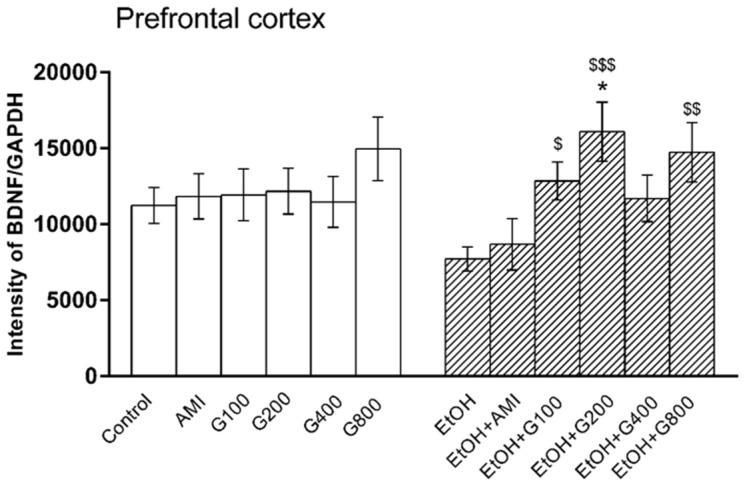
Effects of treatments on BDNF levels in the prefrontal cortex. AMI, amitriptyline; EtOH, ethanol; G100, G200, G400, G800, ginseng extract G115 100, 200, 400, or 800 mg/kg, respectively. * *p* < 0.05 compared to Control, ^$^ *p* < 0.05, ^$$^ *p* < 0.01 and ^$$$^ *p* < 0.001 compared to the EtOH group.

One-way ANOVA showed the effect of treatment on BDNF levels in the prefrontal cortex (*F*(11,73) = 2.17, *p* = 0.02). Amitriptyline and G115 did not change BDNF levels in the prefrontal cortex of normal mice compared to controls. BDNF levels in the prefrontal cortex of mice in the ethanol-treated group tended to decrease compared to controls, but this was not significantly different. However, mice receiving G115 (100, 200, and 800 mg/kg) plus ethanol had significantly higher levels of BDNF in the prefrontal cortex compared to that of mice in the ethanol-treated alone group (*p* < 0.05, *p* < 0.001, and *p* < 0.01, respectively). Moreover, mice receiving G115 (200 mg/kg) plus ethanol had significantly higher BDNF levels in the prefrontal cortex compared to controls (*p* < 0.05) (Figure 4).

## 4. Discussion

Treatments of depression symptoms in alcohol use disorder are needed to support alcohol abstinence and prevent relapse. The effective drug to treat this condition requires further investigation. Ginseng and ginsenosides have previously shown their benefits in various neurologic and psychiatric disorders, clinically and in animal models [15]. Herein, we provide the evidence of the antidepressant-like effect of ginseng extract G115 in normal and ethanol-induced depression in mice. The antidepressant mechanism of ginseng extract G115 involves the increase of BDNF levels in the hippocampus and prefrontal cortex. 

Previous studies reported the antidepressant-like effect of ginseng total saponin and ginsenosides in various animal models, including chronic mild stress, olfactory bulbactomy and corticosterone-induced depression [18,19,20,21]. The present study demonstrated the antidepressant-like effect of ginseng extract G115 in normal mice and, importantly, in ethanol-treated mice using the forced swimming test. This effect of ginseng extract G115 is comparable to amitriptyline, an antidepressant used as a positive control in this study. Moreover, the potent effect of various doses of G115 (100, 200, and 800 mg/kg) is observed as changes of immobility time and swimming time in mice receiving these doses plus ethanol were also significantly higher than those in controls. 

The dose-independent effect of G115 in this study was noticeable as G115 100 and 800 mg/kg produced higher antidepressant effects than G115 200 and 400 mg/kg. Previous study also reported the dose-independent antidepressant effect of ginsenoside Rb3 [22]. Moreover, the synergistic effect and interaction of active compounds in the herbal extracts, for example Rhizoma coptidis and Salvia miltiorrhiza, have been reported [23]. As G115 is composed of various ginsenosides, the interaction of the compounds might lead to the increase or decrease of pharmacological responses observed in this study.

BDNF is essential for neuronal plasticity and survival in the adult brain [9]. Various neurotoxins, including ethanol-decreased BDNF levels and expression resulting in the impairment of neuronal differentiation and neuronal death. Thus, the reduction of brain BDNF has been suggested as one of the mechanisms of alcohol-induced depression [7,8,12]. The present study revealed that repeated ethanol administration caused depression-like behaviors together with the subtle reductions of BDNF levels in the hippocampus and prefrontal cortex. Although these reductions were not significantly different from controls, the reduction of brain BDNF levels are partly responsible for ethanol-induced depression.

An increase of brain BDNF levels is one of the proposed mechanisms of antidepressants. Chronic fluoxetine treatment increased brain BDNF mRNA expression while repeated administration of imipramine protected against the stress-induced decreased of BDNF [24,25]. Moreover, BDNF infusion into the brain can produce an antidepressant effect [26]. Ethanol serves as the stress in this study. Previous studies demonstrated that chronic stress could lead to BDNF downregulation and BDNF plays a key role in depression recovery by promoting neuronal plasticity [27,28]. The present study showed no, or little, effect of G115 on BDNF levels in normal mice performing a forced swimming test. However, G115, as well as amitriptyline, markedly increased brain BDNF levels in ethanol-treated mice. G115 promoting BDNF augmentation in ethanol-treated mice might support the survival and plasticity of neurons during stress-induced depression leading to the antidepressant effect of G115.

Alterations of brain BDNF levels in the pathophysiology of depression and the effects of antidepressants depend on brain areas [29]. An increase of the hippocampal BDNF following chronic antidepressant treatment is associated with neurogenesis [30], while the antidepressant action in the prefrontal cortex relates to synaptic plasticity [31]. In this study, the potent effect of G115 and amitriptyline on BDNF levels was observed in the hippocampus, but the modest effect was revealed in the prefrontal cortex. The regional-specific effects of antidepressants on brain BDNF levels were previously reported [24,25,32]. The increase of neurogenesis in the hippocampus is one of the neuroprotective mechanisms of ginsenoside Rg1 and Rd [19,33,34]. Therefore, the prominent effect of G115 in the hippocampus might involve neurogenesis. However, the effect of G115 in neurogenesis and synaptic plasticity should be further investigated.

## 5. Conclusions

The present study demonstrates the antidepressant-like effect of ginseng extract G115 in normal and ethanol-treated mice. The mechanism involves an augmentation of BDNF levels in both the hippocampus and prefrontal cortex. These findings provide scientific information and support the benefit of using ginseng extract G115 for the treatment of depression in alcohol use disorder. 

## Figures and Tables

**Table 1:** Treatment groups. Water, ethanol, and amitriptyline were given 10 mL/kg, 3 g/kg, and 15 mg/kg, respectively. Mice received p.o. treatment one hour prior to i.p. treatment.

Groups	Treatment	Groups	Treatment
Normal Mice		Ethanol-Treated Mice	
1. Control	Water p.o. + Water i.p.	7. EtOH	Water p.o. + Ethanol i.p.
2. AMI	Amitriptyline p.o. + Water i.p.	8. EtOH + AMI	Amitriptyline p.o. + Ethanol i.p.
3. G100	G115 100 mg/kg p.o. + Water i.p.	9. EtOH + G100	G115 100 mg/kg p.o. + Ethanol i.p.
4. G200	G115 200 mg/kg p.o. + Water i.p.	10. EtOH + G200	G115 200 mg/kg p.o. + Ethanol i.p.
5. G400	G115 400 mg/kg p.o. + Water i.p.	11. EtOH + G400	G115 400 mg/kg p.o. + Ethanol i.p.
6. G800	G115 800 mg/kg p.o. + Water i.p.	12. EtOH + G800	G115 800 mg/kg p.o. + Ethanol i.p.

AMI, amitriptyline; EtOH, ethanol; G100, G200, G400, G800, ginseng extract G115 100, 200, 400, or 800 mg/kg, respectively.
